# Dexmedetomidine as Conduit for Non-Invasive Ventilation (NIV) Compliance in COVID-19 and Chronic Obstructive Pulmonary Disease (COPD) Patients in Intensive Care Unit (ICU) Setting: Case Series

**DOI:** 10.7759/cureus.33981

**Published:** 2023-01-19

**Authors:** Mohammad H Akhtar, Shahla Haleem, Nazia Tauheed, Deeba Khan

**Affiliations:** 1 Department of Anesthesiology, Jawaharlal Nehru Medical College, Aligarh Muslim University, Aligarh, IND

**Keywords:** sedation in icu, rass score, oxygenation, niv compliance, dexmedetomidine, copd, covid-19

## Abstract

Non-compliance to the non-invasive ventilation (NIV) mask in a distressed hypoxemic patient is not an unusual finding, especially in desaturated coronavirus disease (COVID-19) or chronic obstructive pulmonary disease (COPD) patients with respiratory distress who require ventilatory support to improve oxygenation. Failure to achieve success with the non-invasive ventilatory support with the tight-fitting mask led to emergent endotracheal intubation. This was in view to avert consequences such as severe hypoxemia and subsequent cardiac arrest.

Sedation is an important component of ICU management for noninvasive mechanical ventilation to improve NIV compliance/tolerance. Including the various sedatives used, such as fentanyl, propofol, or midazolam, the most suitable agent to be used as a primary/sole sedative still remains unclear. Dexmedetomidine providing analgosedation without significant respiratory depression facilitates better tolerance of NIV mask application. This case series is a retrospective analysis of patients in whom dexmedetomidine bolus followed by infusion was observed to facilitate compliance to NIV with the tight-fitting mask. Herein, a case summary of six patients with acute respiratory distress who were dyspnoic, agitated have severe hypoxemia were put on NIV with dexmedetomidine infusion is being reported. They were extremely uncooperative as their RASS score (Richmond Agitation-Sedation score) was + 1 to +3, not allowing the application of the NIV mask. Due to their poor compliance with to use of the NIV mask, proper ventilation could not be achieved. Dexmedetomidine infusion (0.3 to 0.4 mcg/kg/hr) was used after a bolus dose (0.2-0.3 mcg/kg). The RASS Score of our patients was +2 or +3 before this intervention which became -1 or -2 after including dexmedetomidine in the treatment protocol. The low dose dexmedetomidine bolus and infusion thereafter showed to improve the patient’s acceptance of the device. Oxygen therapy with this was shown to improve patient oxygenation by allowing the acceptance of the tight-fitting NIV face mask. In conclusion, this case series serves as evidence of the use of dexmedetomidine as an effective therapy to calm the agitated desaturated patient, thereby facilitating non-invasive ventilation in COVID-19 and COPD patients and promoting better oxygenation. This may, in turn, avoid endotracheal intubation for invasive ventilation and the associated complications.

## Introduction

According to the official report on the COVID-19 page of the World Health Organization, globally, as of 5:34 pm CET, 1 December 2022, there have been 639,572,819 confirmed cases of COVID-19, including 6,615,258 deaths, reported to WHO. While early intubation was the initial recommended strategy for COVID-19 patients with severe hypoxemia, a large case series in the US, as well as data from Britain, China, and Italy, suggests high mortality for patients requiring invasive ventilation [1]. The National Institute of Health now recommends high-flow nasal cannula (HFNC) as first-line oxygen support [2]. The COVID-19 clinical spectrum ranges from asymptomatic cases to severe respiratory involvement leading to hypoxemia and multiple organ dysfunction syndrome. Various oxygen therapy modalities have been used in such patients, including invasive and non-invasive mechanical ventilation.

To overcome the hypoxemic crisis, immediate oxygenation is mandatory for these patients. This is not usually feasible in a distressed, anxious patient without resorting to invasive ventilation. Non-invasive ventilation strategies are not well tolerated. Dexmedetomidine due to its sedative, analgesic, and anti-delirium effects, without respiratory depression, may serve as a conduit for acceptance of NIV mask for non-invasive ventilation in a non-compliant, uncooperative patient, leading to effective ventilation and even avoid endotracheal intubation with its consequences as reported by Karim et al. [3]. Recently, for sedation in Intensive Care Unit (ICU), it emphasized maximizing patient comfort so that they remain cooperative, oriented, and able to follow instructions. This is observed to be feasible with dexmedetomidine under targeted therapy to a desired sedation of 0 to -2 RASS score [4].

## Materials and methods

Case report

We reported six patients with acute respiratory distress, five cases were of COVID-19, and one was of acute exacerbation of COPD. All were severely hypoxaemic, dyspnoic, and in an agitated state at the time of admission. The criteria for acute respiratory distress was taken as a PaO2/FiO2 ratio of less than 200 mm Hg. The patients who were admitted to the intensive care unit (ICU), requiring NIV, were uncooperative, rated as +1, +2, or at times even +3 on the Richmond Agitation-Sedation Scale (RASS). The dexmedetomidine was administered intravenously, 0.2 to 0.3 mcg/kg as a bolus in 10-15 minutes, followed by 0.3-0.4 mcg/kg/hr infusion. The RASS is a structured assessment system for the evaluation of sedation and agitation; sedative medications may be titrated as per the desired target scoring [3]. It is a 10-point scale describing various levels of agitation/sedation, with 0 representing the awake and calm patient, +4 (overtly combative) to +1(restless), and −1(drowsy) to −5 (unarousable). The target sedation level was kept as a RASS of (-1) or (-2) for patients included. 

In COVID-19, dexmedetomidine was given as a trial in our ICU, where commonly used sedatives such as haloperidol, fentanyl, midazolam, and promethazine turned out to be ineffective. 

However, this medication was not used in patients who are gasping or in severe respiratory distress warranting immediate endotracheal intubation (who cannot be put on an NIV face mask) or those with hemodynamic instability, hepatic or renal failure; GI bleed was excluded to give dexmedetomidine for sedation. Informed consent was obtained from the patient’s relative before administering the drug, the patient being too agitated/delirious. During administration, patients were monitored for RASS scoring, apart from the basic monitoring of blood pressure (BP), heart rate, respiratory rate, complications associated with sedation, and other parameters as indicated by the patient’s clinical condition. Weaning from NIV was planned once there was no dyspnoea at inhaled oxygen percentage (FiO2) of 40% either on CPAP (4 cm) mode or Bi level-PAP mode; the Pao2 should be at least 100 mmHg at room air.

Case 1

A 52-year male, weighing 65 kg, and COVID-19 positive, with a history of systemic hypertension for the past 20 years, was brought to our facility with a complaint of mild to moderate fever for the last 10 days along with shortness of breath, which increased progressively to such an extent that now patient had dyspnoea at rest. On examination, the patient had a respiratory rate (RR) ≥ 30 breaths per minute, oxygen saturation (SpO2) was ≤ 74% on room air, and auscultation revealed bilateral crepitations and > 50% lung infiltrates on the X-ray chest. Therapy was immediately begun with oxygen administration with a high flow nasal cannula (HFNC), iv antibiotics, and steroids, targeting a peripheral oxygen saturation as measured by a pulse oximeter (SpO2) of 92%. Oxygen saturation failed to improve even on high-flow oxygen; the patient was then shifted to ICU for mechanical ventilation. Non-invasive bi-level positive pressure (BiPAP) ventilation was given a trial through a non-vented face mask, settings kept as FiO2= 50%, I/E PAP = 10/5 cm H2O, and R/R of 15 breath/min. But the patient was very much agitated and unable to tolerate the mask. Inj. haloperidol and inj. promethazine was given intramuscularly, but no success could be achieved in calming down the patient. Dexmedetomidine 0.2 mcg/kg intravenous bolus followed by infusion at 0.4 mcg/kg/hr was started. This helped the patient to calm down and use the NIV mask for longer periods, improving ventilation and oxygenation. The patient was kept on continuous NIV with good acceptance, later on intermittently. He improved and was weaned off subsequently in the next five days.

Case 2

A 68-year male without any comorbidities reported to the emergency with complaints of cough and shortness of breath for the last two days. His oxygen saturation was 82% on room air. Oxygen administration was begun through Hudson mask, along with intravenous (i.v.) antibiotics and steroids. Improvement was seen initially for the first few days, but the patient again started complaining of shortness of breath, and his oxygen saturation started falling despite increasing inhaled oxygen delivery. As he was COVID -19 positive, invasive mechanical ventilation was planned to be avoided. He was kept on non-invasive ventilation, but he was repeatedly removing the NIV mask; thus, oxygenation could not be achieved adequately. Dexmedetomidine 15mcg bolus followed by infusion at the rate of 0.3mcg/kg/hr was started. Thereafter, the patient was able to breathe properly, his hemodynamics settled, and adequate oxygenation could be achieved. 

Case 3

A 40-year female who was a known case of diabetes mellitus, hypothyroidism and COPD reported to the emergency with complains of shortness of breath, intermittent fever, and cough for the past 15 days. On presentation, her oxygen saturation was 70% on room air with bilateral fine crepitations on chest auscultation. Chest X-ray revealed bilateral bronchopneumonia. Oxygen therapy was initiated immediately with Hudson’s mask at 8-10 L/min, patient improved transiently. Over the next two days, her oxygen requirement increased, followed by increased respiratory rate and labored breathing. She was shifted to ICU for further management. She was initiated on bi-level positive airway pressure (BiPAP) support using a non-vented face mask. Initially, the patient had good acceptance of the NIV mask with intravenous inj. fentanyl and midazolam. The patient-reported discomfort, and her oxygen requirement increased. A feeling of suffocation on the NIV mask was reported, which led to removing the mask (NIV failure). To increase the acceptance of face masks and to alleviate anxiousness inj. dexmedetomidine was started at a dose of 0.2mcg/kg/min bolus followed by an infusion, titrated to a dose of 0.45 mcg/kg/hr to achieve a state of calmness and comfort in the patient. This aided in the acceptance of the NIV mask and BiPAP ventilation, with improved hemodynamic stability accompanied by improved oxygen saturation and PaO2 in arterial blood gas (ABG) analysis. The patient was later weaned from non-invasive ventilation and finally discharged from the hospital.

Case 4

A 50-year male with a history of fever, difficulty in breathing, and cough for the last six days was brought to our emergency. On arrival, the patient was conscious, oriented, and dyspnoic, with a respiratory rate of 28 breaths per minute and an oxygen saturation of 68% on room air. High-flow oxygen therapy was started. Chest X-ray revealed bilateral bronchopneumonia, and on high-resolution computed tomography (HRCT), the ‘CT severity’ score was found to be 14/25. The patient was shifted to ICU on the third day of emergency admission as he required NIV support. We started BiPAP, but he was restless and had poor acceptance of NIV, which also resulted in anxiety and tachycardia. He was initially given inj. fentanyl and midazolam infusion for NIV compliance, but after the second day, he again became agitated and started removing the NIV mask. Dexmedetomidine was started as a bolus (0.3mcg/kg) and infusion (0.5mcg/kg/hr) intermittently during the use of NIV. This helped in achieving an increased duration of use of BiPAP with decreased agitation and better compliance with the NIV. It also helped in stabilizing the hemodynamic parameters and also relieved the discomfort during NIV use. This translated to improved ventilation and oxygenation, and the general condition of the patient was finally weaned off from NIV after two days of dexmedetomidine infusion.

Case 5

A postpartum day five female with a previous history of peripartum cardiomyopathy developed a fever followed by cough and mild difficulty in breathing. She started oral paracetamol, antibiotics, and oral steroids at home. The patient was relieved from the above complaints but again had a bout of fever on postpartum day 11, which was associated with a fall in oxygen saturation to 89%, and was brought to the emergency department for medical management. She was started on supplemental oxygen via a high-flow nasal cannula for respiratory support. The patient's condition worsened drastically over the next few hours, and her oxygen saturation fell to 68% despite a high flow of oxygen. Though, her HRCT revealed a CT severity score was 7/25. She was brought to ICU immediately and was put on non-invasive ventilation. The patient was very agitated and repeatedly removed her NIV mask because of the feeling of suffocation and discomfort and an inability to sleep. She was given oral lorazepam to help her calm down but to no effect. She was then started on injection dexmedetomidine which was given as 10 mcg iv bolus followed by infusion at 0.3 mcg/kg/hr. This helped in making the patient feel at ease and calming her down and able to feel comfortable to take rest. This also allowed for the increased duration and better acceptance of the NIV mask, which automatically improved her ventilation. The patient's vital parameters improved over the next week with intermittent NIV and dexmedetomidine use. Her general condition and chest X-ray were improved. She was then tapered off NIV and put on HFNC, which ceased the requirement of sedation.

Case 6

A 54-year male came to casualty in respiratory distress, a known case of chronic obstructive pulmonary disease (COPD) and congestive heart failure exacerbations, as well as bronchopneumonia. His baseline vital signs were a heart rate of 127 beats per minute, tachypnoea with a respiratory rate of 32 breaths per minute, a blood pressure of 158/85 mm Hg, his SpO2 was 74% on room air with bilateral fine crepitations in the chest. Oxygen therapy was initiated immediately by Hudson mask at 8-10 L/min, along with iv antibiotics and steroids.

As the patient was in respiratory distress, non-invasive ventilation was started by BiPAP machine using a non-vented face mask (settings: FiO2= 50%, I/E PAP was 10/5 cm H2O, R/R was 16 breath/min). But the patient was very much agitated and unable to tolerate the mask. To alleviate his agitation, dexmedetomidine was administered at a starting dose of 0.2 μg/kg/min as a bolus, titrated to 0.2-0.45 μg/kg/hr infusion for a sedation scale of up to 3. The response could be seen within half an hour of dexmedetomidine; the patient was no longer agitated. His clinical condition improved dramatically, and oxygen saturation and PaO2 in arterial blood gas (ABG) also became better. His vital signs were stable, without any bradycardia or hypotension. Dexmedetomidine infusion was tapered as the patient’s NIV requirements decreased and were stopped, the patient is taken off from NIV with an improved clinical condition, and SpO2 increased to 97%, respiratory rate returned to normal (18 breaths per minute). The total duration of dexmedetomidine administration for this patient was 48 hours. He was discharged from the hospital on the fifth day.

## Results

These initial six cases of COVID-19 were managed with the help of dexmedetomidine to make feasible the NIV mask acceptance; thus, NIV ventilatory support compliant. The patients had RASS of +3 before the intervention, which became -1 or -2 after dexmedetomidine intervention, with improved oxygenation (SpO2) (Table 1).

**Table 1:** Summary of cases managed with the help of dexmedetomidine for NIV support NIV: Non-Invasive Ventilation, BiPAP: Bilevel Positive Airway Pressure, HFNC: High Flow Nasal Cannula, RASS: Richmond Agitation-Sedation Scale

Sl. No. Cases	Age/sex	Clinical Scenario	Ventilatory mode	Drugs initially tried	Second line Drug	Remark
1	51 yrs./M	SpO2=74%, R/R= 30 Hypertensive, COVID positive, acute respiratory failure RASS Score +3	NIV bi-level PAP	Inj haloperidol and inj promethazine	Dexmed 0.2 mcg/kg bolus followed infusion 0.4mcg/kg/hr intermittently	oxygenation improved Spo2=>90%,R/R=16, RASS Score became-2, weaned off on day 5
2	68 yrs./M	SpO2=82%, COVID positive, respiratory distress, RASS Score +3	BiPAP ventilation with orofacial mask	Inj haloperidol and inj promethazine	Dexmed 15 mcg bolus followed infusion 0.3mcg/kg/hr intermittently	Oxygenation improved (Spo2=>92%), RASS Score became-2, weaned off
3	40 yrs./F	SpO2=70%, acute respiratory failure, known case of COPD, DM & hypothyroidism RASS Score +3	NIV BiPAP mask ventilation	Inj fentanyl and midazolam for NIV compliance and failure to weaned off	Dexmed 0.2 mcg/kg bolus followed infusion 0.45 mcg/kg/hr	RASS Score became-2, Spo2=>92% weaned off NIV in 2 days
4	50 yrs./M	SpO2=68%, COPD, acute respiratory distress,with bilateral bronchopneumonia, RASS Score +3	NIV BiPAP ventilation	Inj fentanyl and midazolam for NIV compliance for 4 days failure to weaned off	Dexmed 0.2 mcg/kg bolus followed titrated, infusion 0.4 mcg/kg/h for sedation	RASS Score became-2, Spo2=>90% weaned off NIV within 2 days
5	30 yrs./F postpartum day 5^th^	COVID positive, respiratory failure, with peripartum cardiomyopathy Spo2=69% RASS Score +3	HFNC followed by BiPAP NIV ventilation	Inj haloperidol and inj promethazine	Dexmed 10 mcg bolus followed titrated, infusion 0.3 mcg/kg/hr	oxygenation improved, Spo2=>92% RASS Score -1, weaned off on day 2
6	54 yrs./M	COPD, R/R=32/min, Spo2=>68%, bilateral bronchopneumonia in CHF, COVID positive RASS Score +3	NIV BiPAP ventilation	Inj haloperidol and inj promethazine	Dexmed 0.2 mcg/kg bolus followed titrated, infusion 0.2-0.4 mcg/kg/hr	oxygenation Improved, Spo2=>90% RASS Score became-2,

## Discussion

The COVID-19 disease causes influenza-like symptoms ranging from fever, sore throat, body ache, and cough to shortness of breath, fall in oxygen saturation, and even chest pain. Oxygen therapy is indicated in moderate and severe cases through an HFNC, non-invasive ventilation, or an invasive ventilatory support.

Non-invasive ventilation (NIV) is a well-established treatment for acute respiratory failure [1], especially in patients with hypercapnia [2] and cardiogenic pulmonary edema [5]. There is now growing evidence that NIV may be of benefit to patients early in the disease process and may also prevent further deterioration and the need for endotracheal intubation [6]. It acts by supplying a mixture of air and oxygen using positive pressure to help the patient breathe comfortably, therefore, improving oxygenation, increasing the lung volume, and decreasing the work of breathing, although this has little or no effect on the natural course of the disease [7,8].

NIV is delivered via a tight face mask or a helmet. NIV includes both the continuous positive airway pressure (CPAP), in which a constant driving pressure is supplied by the machine, and the bi-level positive airway pressure (Bi-level/BIPAP), in which the driving pressure alters rhythmically between inspiration and expiration [8]. The initiation of breath in BIPAP has to be driven by the patient. Therefore, a conscious patient able to initiate their own breaths and able to maintain their own airway is a key factor in the success of NIV [7,9]. Patient acceptance and compliance/coordination are required to prevent any ventilator asynchrony, which has been found to adversely affect sleep, an established cause of NIV failure [10-12]. 

Sedation has been proposed to help in mechanical ventilation, allaying anxiety, improving sleep, and thereby, helping in modulating physiologic response to stress such as tachycardia and hypertension [13,14]. Various studies have addressed the efficacy of sedation during NIV using dexmedetomidine, midazolam, propofol, and remifentanil in patients with several diseases in which there was a high to intermediate level of evidence for NIV use [11,15-18]. Opioids have been commonly used in mechanically ventilated patients in the ICU, and fentanyl is the most commonly used sedative drug in ICU [19]. Respiratory depression and alertness of an individual to maintain his own airway limits the use of benzodiazepines and opioids as sedatives in ICU.

Ketamine does not cause respiratory depression [20]. It limits airway resistance, thereby improving dynamic compliance, minute ventilation, functional residual volume, and tidal volume without inhibiting protective laryngeal and pharyngeal reflexes. However, it precipitates hypersalivation and emergence reaction along with its relative contraindication in hypertension and those with an increased cardiac workload. Hence, its use in NIV is controversial.

Dexmedetomidine is an α2 adrenoreceptor agonist with potent anxiolytic, sedative, and mild analgesic effects without any respiratory depression. The lack of respiratory depression and arousable sedation makes it a very convenient drug to use in patients with NIV support [21]. The drug has also been known to have both cytoprotective and anti-inflammatory properties [22]. Its organ protective effects against acute organ injury, such as brain, lung, and kidney, have been well established in pre-clinical settings [23,24]. Its cholinergic anti-inflammatory mechanisms are also postulated to suppress excessive inflammatory responses to COVID-19 [25]. Demuro et al., in their study, used dexmedetomidine to improve ventilator synchronization among patients with acute respiratory failure and reduce the length of ICU stay [25]. However, the role of combination therapy for sedation may be a better choice to improve ventilator synchronization, and NIV mask acceptance as a single drug is inadequate to achieve the target sedation [26,27]. The other facts which are relevant in our cases are that - COVID-19 patients are in requirement of higher sedation and frequently require more than one drug. This might be also one of the reasons why our patients failed the first line [27]. Muriel et al. had shown an improved outcome if proper sedation is achieved following non-invasive ventilation [28].

This study entails substantial limitations, a small case series limited to a single center covering a small geographical area. As most of our patient belongs to COVID-19, we failed to get detailed data regarding the lab findings. The data were based on clinical outcomes rather than a methodically well-planned study. With most of the patient population belonging to the lower socioeconomic strata catered by the institute, drug procurement was also a limitation. Large-scale, multi-centered randomized trials are needed to establish the results and findings of this study.

## Conclusions

Dexmedetomidine was found an effective sedative for non-invasive ventilation in COPD/ COVID-19 patients with acute respiratory insufficiency/failure to improve oxygenation, absence of respiratory side effects, prevent ventilator asynchrony, maintain intact airway reflexes, maintain organ perfusion, and prevent the onset of or progression to multi-organ dysfunction and the need for invasive ventilation with its attendant complications was averted. Therefore, we recommend that low-dose dexmedetomidine (0.2 to 0.3 mcg/kg as a bolus followed by 0.3-0.4 mcg/kg/hr infusion) may be used as a sedative of choice in a very agitated and hypoxemic but hemodynamic stable patient who do not tolerate close-fitting NIV face mask, especially in a setting of prior administration of midazolam/ fentanyl/haloperidol/phenothiazine, as has also been suggested recently.
